# A rare primary *Candida parapsilosis* infection of the knee joint in a patient without predisposing factors: A case report

**DOI:** 10.1097/MD.0000000000014327

**Published:** 2019-02-08

**Authors:** Jun Wang, Zhen Zhang, Miao Zhang, Bo Yang, Tengyun Wang, Xuedong Sun, Xiuli Chen, Min Yi Zhang, Zhi Yong Guo, Xin Jiang

**Affiliations:** Department of Joint Surgery, Weifang People's Hospital, Shandong, China.

**Keywords:** *Candida parapsilosis*, infection, knee joint

## Abstract

**Rational:**

Knee joint infection caused by isolated primary *Candida* is extremely rare, with only a few cases reported. It occurs most often in patients with predisposing factors (e.g., immunosuppression, malignancy, drug abuse) or fungal invasion during traumatic procedures, including surgery. We report an unusual case of *Candida parapsilosis* infection in the knee joint with no predisposing factors.

**Patient concerns:**

A 65-year-old man entered our hospital complaining of persistent pain and mild swelling of the right knee that seriously affected normal walking. There was no obvious cause for his distress.

**Diagnosis:**

The case was eventually diagnosed as a primary *Candida parapsilosis* infection which had many diagnostic particularities and difficulties.

**Interventions:**

Total knee arthroplasty (TKA) was ultimately performed followed by fluconazole coverage.

**Outcomes:**

The patient showed good clinical performance at the 3- and 6-month follow-up visits and was very satisfied with the therapeutic effect.

**Lessons:**

If there were suspected symptoms of primary *Candida* infection cases, imaging and microscopic examinations, tissue cultures, and pathological examination of the puncture knee joint fluid were required.

## 1 Introduction

*Candida* infection of the knee joint is rare, and primary knee joint candidiasis is extremely rare, with only a few cases reported. In most cases, arthritis due to *Candida* is caused by hematogenous dissemination. The most common cause of *Candida* infection is accidental implantation of the fungus during the course of trauma treatment, such as by intra-articular injections, surgery, prosthesis implantation, the spread of infection in adjacent areas, and immunosuppression such as in human immunodeficiency virus (HIV)-infected people with intravenous drug use.^[1–5]^ Although some *Candida* infections (e.g., *C Lusitaniae*, *C tropicalis*, *C albicans*) have been reported to be the causes of joint infection.^[6–10]^ Until now, however, primary *Candida parapsilosis* had never been reported to be the cause of a knee joint infection.

We report a patient with primary *C parapsilosis* infection in the knee joint without predisposing factors who was successfully treated with total knee arthroplasty (TKA) and fluconazole. As far as we could determine, this is the first reported case of a primary *C parapsilosis*-related knee joint infection. This case was originally misdiagnosed as severe osteoarthritis, for which the patient underwent TKA. Afterward, the knee was found to be infected by primary *C parapsilosis* and was successfully treated and cured by fluconazole administration. We believe that by reporting the clinical characteristics, detailed diagnosis, and treatment of the patient, combined with a review of the literature, perhaps we can help provide early correct diagnoses and treatment of similar clinical cases.

## 2 Case report

A 65-year-old man came to our hospital with an 8-year history of pain and swelling of the right knee, with the pain particularly aggravated for the past 4 years. The pain increased with exertion and was relieved with rest. During the past 4 years, the pain markedly increased, and the joint had repeated bouts of swelling. The patient had been treated with oral anti-inflammatory and analgesic drugs, with little effect. He came to our hospital for further treatment and was diagnosed with severe osteoarthritis of the knee based on the radiologic and physical examinations. It was decided to perform TKA.

**Figure 1:**
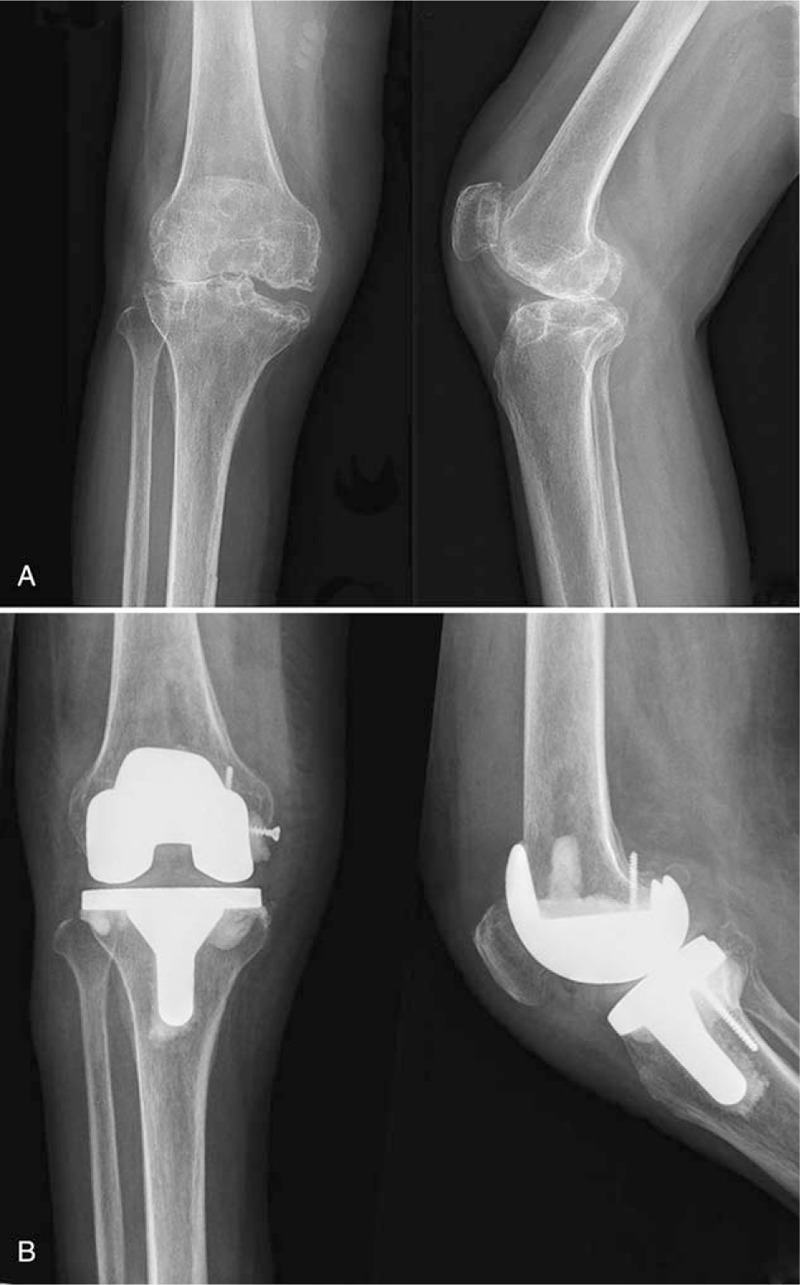
A, X-ray showed severely degenerative and osteophyte formation. B, The prosthesis in good position.

There was no history of rheumatoid disease, cancer, kidney disease, tuberculosis, HIV infection, or hepatitis. The patient denied a history of smoking, drinking, steroid use, and illegal drug abuse. The family and psychosocial histories were insignificant. It was important that the patient had had no previous knee puncture or knee trauma. Physical examination revealed mild knee swelling and pain, but the local skin temperature was normal. Knee radiographs revealed bone damage in the distal femur and proximal tibial subchondral bone, serious joint space narrowing, and obvious osteophyte formation (Fig. 1A)—findings that clearly suggested severe osteoarthritic changes in the knee joint.

**Figure 2:**
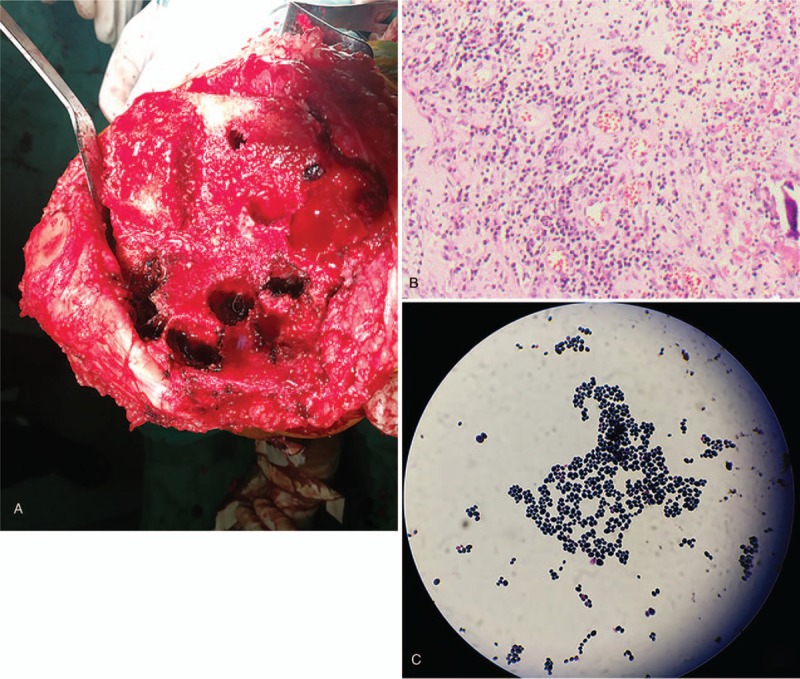
A, Several subcartilaginous focal cavities were seen in the operation. B, Pathological results showed that mild inflammationeration. C, Tissue culture suggested that *Candida parapsilosis* infection.

On admission, laboratory studies revealed the following: white blood cell count 9.29 × 10^9^ (4–10 × 10^9^); erythrocyte sedimentation rate (ESR) 7 mm/h (0–15 mm/h); C-reactive protein (CRP) 4.9 mg/L (0–8.0 mg/L); and parathyroid hormone 37.05 pg/mL (15.00–65.00). Tests for antinuclear antibody, rheumatoid factor, anti-streptolysin O, and HLA B27 were all negative. Three days after admission, TKA was performed. During the operation, after cutting the bone we found several small, focal cavities under the cartilage. We filled them with bone cement with added vancomycin and implanted screws (Fig. 1B). During the surgery, we also found mild synovial inflammation and focal cystic degeneration of bone in the knee joint (Fig. 2A).

Postoperatively, we performed bacterial and fungal cultures and pathology examinations. The postoperative pathology results showed synovial tissue hyperplasia accompanied by mild inflammation, as well as tissue degeneration with nuclear fragments (Fig. 2B). Postoperative tissue cultures were negative for bacteria, but the fungal culture results suggested *C parapsilosis* infection (Fig. 2C).

Based on the results of the postoperative drug susceptibility tests, the patient was given fluconazole (400 mg/d i.v.). After 6 weeks, the fluconazole was discontinued, and the patient was discharged from the hospital. We asked the patient to return to the hospital for review at 3 and 6 months after the operation. He complied, and at 6 months there were no signs of recurrent infection.

Ethical approval was obtained from the Ethics Committee of Weifang People's Hospital and the patient gave his written informed consent and the ethics committee approval number was LL-2018-012.

## 3 Discussion

In recent years, there has been an increasing number of *C parapsilosis* infections reported, with their pathogenesis associated with many factors, included adhesion, biofilm formation, and hydrolytic enzymes. Hydrolase has been an important virulence factor and has played an important role in the invasion process. In Latin America, Canada, Europe, and the Asia-Pacific region, *C parapsilosis* is now the second-most invasive *Candida* species, exceeded only by *C albicans*. Data from China's CHIF-NET supports these figures.^[11–14]^

*C parapsilosis* is widely distributed in nature, and blood is its most common means of spread although it has also been isolated from ascites fluid, synovial fluid, urine, cerebrospinal fluid, and other bodily fluids. There are many other factors favoring *C parapsilosis* infection, including catheter implantation (97%), broad-spectrum antibiotic application (91%), total parenteral nutrition (54%), abdominal surgery (46%), glucocorticoid use (38%), tumors (27%), organ transplantation (16%), granulocyte deficiency (12%), and previous *Candida* colonization (11%).^[15]^

The literature has shown that almost all persons with *C albicans* infection have low immune function or damaged tissues due to the presence of underlying diseases and sometimes secondary to trauma and surgery. In our case, however, there were no predisposing factors for *Candida* infection, and the patient had no history of a previous knee puncture or knee trauma.

Most clinicians confronted with a knee infection suspect pyogenic activity or *Mycobacterium tuberculosis* infection. They may not consider a fungal infection because of its low incidence.^[16]^ As far as we know, fewer than 10 reported knee joint infections have been associated with *C albicans*, which has been found mainly in newborns, drug abusers, HIV-infected persons, and others.^[17]^ We found only 1 case of primary *Candida* infection in the knee joint. In that case, the physician performed TKA, during which severe inflammation and abscesses were found. The operation was therefore suspended and anti-inflammation treatment performed. After the inflammation resolved, TKA was undertaken, but a skin infection occurred 1 week after the operation.^[18]^

Fungal infections often manifest as systemic symptoms, so early diagnosis and treatment should be performed. Primary candidiasis of the knee joint, however, usually has a long, chronic course, often without systemic symptoms. In our case, we found that the routine blood tests (e.g., ESR, CRP) were normal. Hence, if there is only mild knee pain and swelling, and antibiotic treatment is ineffective but more knee swelling and pain occurs—and if radiographs reveal focal cystic degeneration under the cartilage—it must be considered that primary *Candida* infection may be present. In such a case, the physician should conduct a careful physical examination, computed tomography evaluation, prepare bacterial and fungal cultures, and carefully examine the joint fluid.

There is limited information on the treatment of knee infections caused by primary *C albicans.* Fluconazole has proved successful for treating primary *Candida* infections.^[19]^ There are reports that both amphotericin B and fluconazole could be used to control *Candida* infections before TKA.^[18]^ In our case, the preoperative examination of the patient was normal, and we misdiagnosed the case as severe osteoarthritis and undertook TKA. During that operation, we found that there were several sites of focal cystic degeneration under the cartilage, so we thoroughly cleaned the lesions and implanted vancomycin-synthesized bone cement to plug the cavities before proceeding with TKA. It was the routine postoperative tissue cultures that revealed the infective organism was *C parapsilosis*.

A long-term survey of *Candida* showed that fluconazole has good antifungal effects against it and few side effects.^[20]^ Therefore, we applied a 6-week intravenous infusion of fluconazole with periodic review of liver and kidney functions. At both 3 and 6 months after discontinuing the fluconazole, the patient had no inflammation and no signs of infection.

Although a case review found that fungus-related knee infections are rare but often cause systemic symptoms, it was also found that primary *C parapsilosis* infections generally produce no systemic symptoms or only mild swelling and little pain in the knee joint. Nevertheless, the destructive consequences are often irreversible and so require close attention. In our case, the preoperative examination of the patient was normal, so we wrongly performed TKA. It was only after that surgery that *C parapsilosis* was discovered in a routine tissue culture. We applied fluconazole as a remedy, and fortunately the final effect was good.

We learned much during this clinical experience. First, if you suspect a primary *Candida* infection of the knee joint, conduct a comprehensive examination to confirm the diagnosis, including detailed blood examination, radiographic examination, and knee joint punctures to acquire joint fluid for cultures. After the diagnosis is confirmed, treatment should be started with fluconazole to address the infection. Once the infection disappears, TKA can be performed. We also discussed whether TKA is feasible under the condition when only the routine blood tests (e.g., ESR, CRP) are normal. This decision requires an extreme depth of clinical practice to get it right.

## 4 Conclusion

Primary *Candida* infection of the knee joint is extremely rare. In addition, the infection often has no systemic symptoms, only slight joint swelling and pain. If it is not diagnosed and treated early, however, the consequences often lead to irreversible bone destruction and joint deformity. If the patient complains of slight pain and swelling in the knee, and there has been a long, unsuccessful course of treatment with antibiotic therapy, radiography may reveal the formation of bone cavities under cartilage. In such cases, consider the possibility of a fungal infection and then obtain more computed tomography scans and cultures of knee joint fluid. Bone cement mixed with vancomycin, fluconazole, and TKA were effective for treating knee joint infections caused by primary *C parapsilosis*.

## Acknowledgments

The authors thank all of nurses and personnel of Weifang People's for their cooperation in all the stages of the study.

## Author contributions

**Data curation:** Zhen Zhang.

**Formal analysis:** Bo Yang, TengYun Wang.

**Investigation:** XueDong Sun, XiuLi Chen, Xin Jiang.

**Software:** Miao Zhang.

**Supervision:** Zhi Yong Guo.

**Writing – original draft:** Jun Wang.

**Writing – review & editing:** Min Yi Zhang.
